# Influence of Microgravity on Apoptosis in Cells, Tissues, and Other Systems In Vivo and In Vitro

**DOI:** 10.3390/ijms21249373

**Published:** 2020-12-09

**Authors:** Binod Prasad, Daniela Grimm, Sebastian M. Strauch, Gilmar Sidnei Erzinger, Thomas J. Corydon, Michael Lebert, Nils E. Magnusson, Manfred Infanger, Peter Richter, Marcus Krüger

**Affiliations:** 1 Gravitational Biology Group, Department of Biology, Friedrich-Alexander University, Staudtstraße 5, 91058 Erlangen, Germany; binod.prasad@fau.de (B.P.); michael.lebert@fau.de (M.L.); 2 Department of Biomedicine, Aarhus University, Høegh-Guldbergsgade 10, 8000 Aarhus C, Denmark; dgg@biomed.au.dk (D.G.); corydon@biomed.au.dk (T.J.C.); 3 Department of Microgravity and Translational Regenerative Medicine, Clinic for Plastic, Aesthetic and Hand Surgery, Otto von Guericke University, 39106 Magdeburg, Germany; manfred.infanger@med.ovgu.de (M.I.); marcus.krueger@med.ovgu.de (M.K.); 4 Research Group “Magdeburger Arbeitsgemeinschaft für Forschung unter Raumfahrt- und Schwerelosigkeitsbedingungen” (MARS), Otto von Guericke University, 39106 Magdeburg, Germany; 5 Postgraduate Program in Health and Environment, University of Joinville Region, Rua Paulo Malschitzki, 10 - Zona Industrial Norte, Joinville, SC 89219-710, Brazil; sebastian.michael@univille.br (S.M.S.); gerzinger47@gmail.com (G.S.E.); 6 Department of Ophthalmology, Aarhus University Hospital, Palle Juul-Jensens Blvd. 99, 8200 Aarhus N, Denmark; 7 Space Biology Unlimited SAS, 24 Cours de l’Intendance, 33000 Bordeaux, France; 8 Diabetes and Hormone Diseases, Medical Research Laboratory, Department of Clinical Medicine, Faculty of Health, Aarhus University, Palle Juul-Jensens Boulevard 165, 8200 Aarhus N, Denmark; nm@clin.au.dk

**Keywords:** microgravity, cosmic radiation, spaceflight, astronauts, mice, rats, cells, tissues, apoptosis

## Abstract

All life forms have evolved under the constant force of gravity on Earth and developed ways to counterbalance acceleration load. In space, shear forces, buoyance-driven convection, and hydrostatic pressure are nullified or strongly reduced. When subjected to microgravity in space, the equilibrium between cell architecture and the external force is disturbed, resulting in changes at the cellular and sub-cellular levels (e.g., cytoskeleton, signal transduction, membrane permeability, etc.). Cosmic radiation also poses great health risks to astronauts because it has high linear energy transfer values that evoke complex DNA and other cellular damage. Space environmental conditions have been shown to influence apoptosis in various cell types. Apoptosis has important functions in morphogenesis, organ development, and wound healing. This review provides an overview of microgravity research platforms and apoptosis. The sections summarize the current knowledge of the impact of microgravity and cosmic radiation on cells with respect to apoptosis. Apoptosis-related microgravity experiments conducted with different mammalian model systems are presented. Recent findings in cells of the immune system, cardiovascular system, brain, eyes, cartilage, bone, gastrointestinal tract, liver, and pancreas, as well as cancer cells investigated under real and simulated microgravity conditions, are discussed. This comprehensive review indicates the potential of the space environment in biomedical research.

## 1. Introduction

Humans in space live in an unusual environment characterized by many stress factors affecting human health. Microgravity, cosmic radiation, vibration, hyper-gravity at launch, and isolation are examples of stress factors that might seriously impact the health of astronauts, cosmonauts, and taikonauts when attending a long-term space mission or exploring outer space [1]. Space travelers have an increased risk of bone loss and muscle atrophy. Other health problems are the initial space adaptation syndrome and motion sickness, slowing of cardiovascular function (hypotension, heart atrophy) together with arrhythmias, changes in the immune system, a decreased production of erythrocytes, and/or ocular disorders [1]. Further symptoms include fluid redistribution (moon-face, puffy face), nasal congestion, and sleeping disorders, among others.

Due to these obvious health problems, it is of great interest to study the impact of microgravity on small animals, tissues, and cells to clarify the underlying mechanisms for these disorders. It is well known that microgravity changes the morphology and growth as well as a large number of biological processes in mammalian differentiated cells and stem cells [2]. Alterations in microgravity-exposed cells, such as differentiation, adhesion, focal adhesion, migration, and proliferation, among other changes, have been reported [3]. An interesting finding in many tissues and benign or malignant cell types exposed to microgravity conditions is an increase in apoptotic cells [4,5,6,7,8,9,10]. Signs of apoptosis have been detected to various extents in cells and tissues exposed to real and simulated microgravity conditions [6,7,9,10,11].

Programmed cell death, or apoptosis, is involved in the pathogenesis of many diseases, such as myocardial ischaemia/infarction, cerebral ischaemia, autoimmune disorders, neurodegenerative diseases, infections, transplant rejection, or in the tumor response to radio/chemotherapy [12]. The process of programmed cell death represents a complex mechanism where a large number of pro-apoptotic and anti-apoptotic factors interact [13]. Apoptosis has important functions in morphogenesis, organ development, and wound healing. In 1972, Kerr et al. [14] described the morphological changes of cells during apoptosis. Shrinkage, chromatin condensation, membrane blebbing, and apoptotic bodies characterize apoptotic cells [14]. In contrast to apoptosis, necrotic areas always show inflammation and oedema, both of which are not found in cells undergoing apoptosis in their environment. 

This review describes the spaceflight options and ground-based techniques suitable for microgravity-based research, the process of programmed cell death, and the current knowledge of apoptosis in small animals, e.g., mice, in space. Available data of the hindlimb suspension (HS) model and the detection of apoptosis in the animals are presented as well. In addition, studies of cells and three-dimensional (3D) tissues or organoids that have been conducted in space or simulated microgravity conditions are illustrated. This review also focuses on the impact of cosmic radiation on cells and tissues. The current knowledge about apoptosis detected in cells from several systems (immune, cardiovascular, gastrointestinal, and nervous), as well as eye cells, bone cells, chondrocytes, and cancer cells, are addressed.

## 2. Spaceflight Options and Ground-Based Techniques Suitable for Microgravity-Based Research

**Figure 1:**
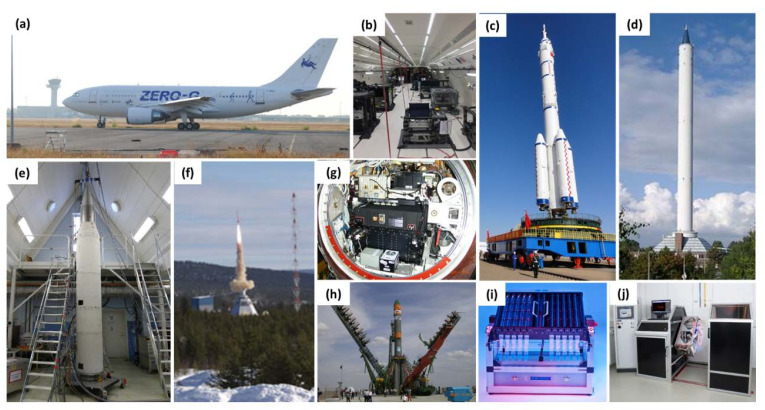
Images of platforms for conducting experiments in real and simulated microgravity conditions. (**a**) Parabolic aircraft (Airbus A310 ZERO G) operated by Novespace, Bordeaux, France. About 22 s of microgravity can be achieved during a parabolic flight manoeuvre. (**b**) Experimental area inside the ZERO G aircraft with different experiment racks. (**c**) The Chinese Long March 2F (Shenzhou 8 mission launched in 2011 from the Jiuquan Satellite Launch Center, China). (**d**) The ZARM drop tower in Bremen, Germany. With a drop tower, approximately 4.5 s of microgravity can be achieved. (**e**) Payload of a MAXUS-sounding rocket (Kiruna, Sweden). A huge castor engine provides sufficient impulse to achieve microgravity up to 14 min. (**f**) Sounding rocket, TEXUS in Kiruna, Sweden, which enables microgravity for about 5–7 min. (**g**) Experiment capsule of the Foton-M No. 2 spacecraft with various experimental containers. (**h**) The Russian Soyuz-U rocket (Foton M No.2-mission, launched in 2005 from the Baikonur Cosmodrome, Kazakhstan). (**i**) Two-dimensional pipette Clinostat (Invented and developed by Jens Hauslage from the DLR. Samples in pipettes are rotated with a speed, which compensates the sedimentation of objects inside the pipettes. Sample fixation is achieved by tilting the rotation platform, so that the samples were simultaneously transferred into flasks with fixation reagent (the falcon tubes on the picture). (**j**) Two-dimensional Clinostat with attached microscope (Fluoreszenz Klinostaten Mikroskop; invented and constructed by Jens Hauslage (DLR) and Kai Waßer (DLR)). The microscope enables bright field as well as fluorescence microscopy. Both the instruments (i, j) are located in the microgravity lab of the DLR in Cologne and are available for external researchers.

Gravity is a permanent environmental stimulus, which cannot simply be switched off or shielded to perform an experiment without its influence on the lab bench. To subject a sample to real microgravity, there are various options, which differ in microgravity duration, quality, and mission preparation time and frequency (Figure 1) [15,16,17,18]. There are notable differences in the time between the final preparation of the experiment and the beginning of microgravity exposure and the time between the end of exposure and recovery of the experiment. In addition, different degrees of automatisation are necessary depending on the applied method to obtain microgravity.

For brief periods, the experimental setup can be put into an airtight capsule and then dropped down in an evacuated tube within a tower, like the drop tower in Bremen, Germany [19]. During the free fall, no acceleration force other than gravity will act within the experiment. The microgravity time is approximately 4.5 s, and at the end of the drop, a massive deceleration occurs during the cushioned impact. The capsule can also be shot from the bottom of the tube upwards, from where it falls back, effectively doubling the microgravity time, but subjecting the experiment to another high-acceleration event [20]. Access to the experimental hardware is possible until a few hours before and after the drop (to allow for the evacuation of the tube before and the equalization of pressure afterwards). Residual vibrations are very low during microgravity, availability is comparatively easy, and the preparation and execution times are rather quick (weeks to a few months). Experiments must be fully automated.

For longer microgravity exposure, parabolic flights can be used [21,22]. The modified airplane climbs at an increasingly steeper angle until 50° and the propulsion is reduced to merely compensate for drag. The airplane and everything aboard now follow a ballistic trajectory (or a parabola), during which microgravity is achieved inside the plane. After passing the peak, the airplane enters a nosedive, which ends at around a −42° decline, and then the airplane is again pulled up to a horizontal flight path. Typically, the microgravity phase lasts for about 22 s and is flanked by two hypergravity phases with about 2 *g* for 20 s each; a flight day consists of 31 parabolas and a campaign lasts for 3–4 flight days. The time between the final experimental preparation on the ground and the first parabola is only a few hours. There are some residual accelerations and/or vibrations during the parabola, depending on flight conditions. The availability is good; European Space Agency (ESA), Centre National D’Etudes Spatiales (CNES) and Deutsches Zentrum für Luft- und Raumfahrt (DLR) organize a total of five to six campaigns per year. The time from proposal to experiment can be as short as a few months. Scientists accompany their experiments aboard, so full automatisation is not required (but highly recommended), and human intervention is possible.

If a longer microgravity exposure is required, a trip to space is unavoidable; the simplest form, a suborbital flight with a sounding rocket, allows for 6 min (TEXUS) or 13 min (MAXUS/MAPHEUS) of microgravity [23]. The sounding rocket propels the scientific payload on a very small parabola up to 260 or 700 km above ground, respectively. Experiments must work fully automatically. After the initial acceleration during launch, residual accelerations are extremely low until landing. The mission preparation time is between one and two years, and access to the experimental hardware integrated in the rocket is possible until 1 h prior to launch. Post-flight recovery might take 2–4 h, or possibly up to 15–20 h, depending on the search/recovery of hardware [23].

For an exposure that lasts a few days, a taxi flight to the International Space Station (ISS) is an option, albeit a rare one [24]. There are satellites equipped with a descent vehicle, a compartment that returns to Earth after a few weeks in Space, like the Russian Bion-M- and Foton-M-type [15] or the Chinese FSW-type [25]. Prior to qualification for manned flight, the Chinese Shenzhou spacecraft was used as an unmanned experimental platform [26]. Flight opportunities are very rare (three in the past 10 years). The satellites are unmanned, and thus experiments must be autonomous. Access to the experiment is allowed until ca. 12–14 h prior to the launch, while access after landing is possible within a few hours. Preparation time requires years. Historically, experiments of comparable duration had been performed on missions of the Space Shuttle (officially, space transportation system (STS)).

The ISS is the microgravity platform with the potentially longest experiment time (several months) [27]. Residual accelerations are very low, and flight opportunities are frequent. Given that crew time is very limited, experiments should be highly autonomous. Due to safety constraints, the design can be quite difficult, and the costs are the highest of all the flight opportunities. The experimental hardware must be delivered days before launch, and between launch and the actual beginning of the experiment, further few days might pass; post-experiment access is possible within days after the end of the experiment [28]. In 2021, the construction of the Chinese Space Station will begin; upon completion, it will offer a potential alternative for long-term experiments in space. 

Access to space microgravity conditions is not an everyday possibility. There are, however, various methods to simulate weightlessness. Results obtained using those methods must be compared with results obtained from experiments in real microgravity to determine the suitability of the method, which depends on the organism involved [29,30,31]. If the direction of the gravity vector is constantly changed, the resulting force over time will become close to zero. For this purpose, the experiment must be rotated, either continuously in one or two axes by means of a two-dimensional (2D)- or 3D-clinostat, or with directional changes by means of a random positioning machine (RPM) [32]. These will achieve the so-called simulated functional weightlessness only in a very small volume in the centre of the rotational axes; hence, they are only suited for very small samples like tissue and cell cultures. Rotating-wall vessels (RWV) are horizontal cylinders that rotate with a speed that compensates the sedimentation speed of the cells inside, simulating weightlessness [33].

Hindlimb suspension (HS) can be used to simulate the effect of microgravity on mammals [34]. The most common technique is to fix the tail of a mouse or a rat to a pulley system installed above the cage. Then, the hind legs of the animal are lifted above the ground, unloading them from any force, while the front legs remain usable for locomotion. After some days of adaptation, the animals behave normally, moving around freely only by means of their front limbs. This simulates the reduced force on the skeleton and muscles of the hind limbs and also promotes cephalic fluid shift. 

The effects of microgravity in humans can be mimicked by head-down bed rest (HDBR) studies [35]. Here, the test person remains on an inclined bed (−6°) with their head slightly lower than their feet for extended periods of time (days to weeks). Many effects of spaceflight on the human body, such as a decrease in bone density, muscle mass, and strength and cephalic fluid shift, are mimicked very well by HDBR [35].

## 3. Apoptosis 

Apoptosis is a form of programmed cell death. It occurs during development when cells are no longer needed, for example, in the metamorphosis of tadpoles [36], or the nematode *Caenorhabditis elegans*. In *C. elegans*, exactly 131 of 1090 cells undergo apoptotic cell death during the ontogenesis of each individual [37]. It is also a defense reaction and can either be triggered by oxidative stress, noxious agents [38], or radiation [39], following a pathway called intrinsic apoptosis. Cells of the immune system can trigger apoptosis in infected cells, a process called extrinsic apoptosis. Over the last two decades, specific pathways leading to regulated cell death were identified and grouped into 12 subroutines [40].

**Figure 2:**
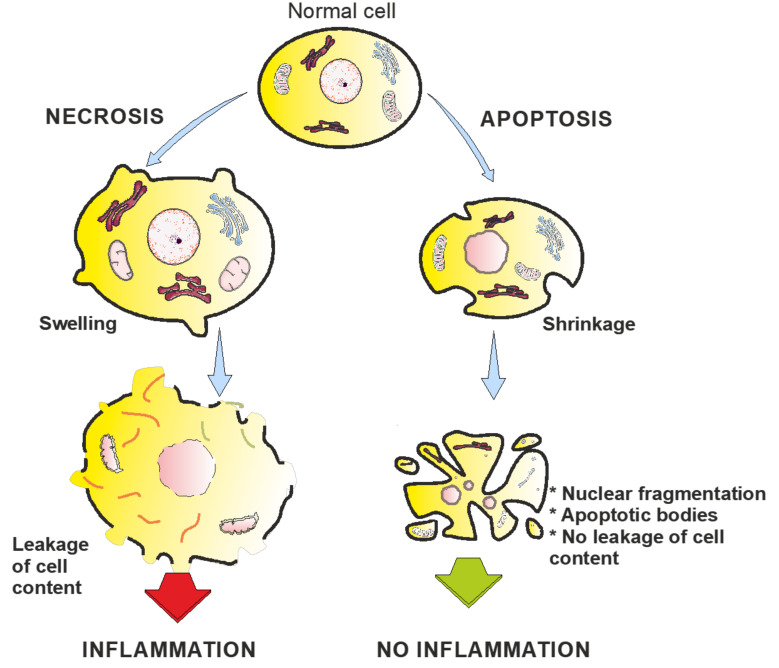
Simplified representation of necrosis and apoptosis in cells.

Apoptosis is a tightly controlled procedure (Figure 2). A generalized process of apoptosis is as follows [41,42]: The cell shrinks and gives up adhesion to surrounding cells, while the cell membrane and the organelles remain intact. Inside the nucleus, the chromatin condenses, a phenomenon called pyknosis. Protuberances occur on the cell surface (blebbing), and the nucleus ruptures into fragments (karyorrhexis). The membrane of the mitochondria ruptures, liberating cytochrome *c*, which in turn triggers a cascade of additional degradation reactions associated with the release of lysosomal enzymes. Finally, small particles enclosed by cell membranes are formed; they contain fragments of the nucleus and organelles, the so-called apoptotic bodies. Surrounding tissues or macrophages quickly absorb the apoptotic bodies to prevent inflammatory reactions within the tissue.

In intrinsic apoptosis, cytochrome *c* is released from the mitochondria into the cytosol, where it forms the apoptosomes with the cytosolic protein apoptosis protease activating factor-1 (Apaf-1) [43]. Apaf-1 subsequently activates the caspase cascade (see below). By contrast, extrinsic apoptosis is a transmembrane-receptor-mediated pathway involving members of the tumor necrosis factor (TNF) receptor gene superfamily [44]. A well-understood model is the TNF-α/TNFR1 reaction: A cluster of three TNFR1 receptors binds to the homologous trimeric TNF-α ligand. The cytosolic end of TNFR1 contains death domains, which are blocked by the inhibitory protein silencer of death domain (SODD). Upon binding of the ligand, SODD dissociates itself and makes room for the adaptor protein TNFR-associated protein with a death domain (TRADD). TRADD in turn recruits Fas-associated protein with a death domain (FADD) and receptor-interacting protein (RIP). FADD binds procaspase-8 and activates it.

Another exemplary system from the TNFR gene superfamily is the Fas receptor/Fas ligand (Fas/FasL) system, which is similar to the TNF-α/TNFR1 system. Upon binding of a FasL to a Fas receptor, a death-inducing signalling complex (DISC) is formed intracellularly, which then activates caspase-8 via FADD. The Fas/FasL system plays a crucial role in the regulation of the immune system [45]. Different cells of the immune system express both Fas and FasL to avoid excessive immune reactions. In addition, some tissues express FasL to counteract lymphocytes and avoid immune intervention, a characteristic called immune privilege [46,47]. Amongst other countermeasures, tumor cells might interfere with the immune reaction by expressing FasL [48].

Caspases (cysteine aspartases) are a family of enzymes that are involved in several kinds of programmed cell death. They are synthesised as inactive procaspases; if triggered, they are activated by (self-)cleaving, which allows for a quick response and regulation. Intrinsic and extrinsic apoptosis pathways merge at the activation of a procaspase. For example, Apaf-1 activates procaspase-9, while FADD activates procaspase-8. Caspases-2, -8, -9, and -10 are initiators, while caspases-3, -6, and -7 are executioners. Initiator caspases activate other caspases, while executioner caspases cut proteins at the site of an aspartate, sometimes with a certain specificity for neighboring amino acids. The destruction of structural proteins, like cytokeratins, leads to the morphological changes typical for apoptosis.

Apoptosis plays a fundamental role in the dynamic equilibrium of bone metabolism. Microdamage or disuse leads to apoptosis of osteocytes. Nearby osteocytes increase the expression of the receptor activator of nuclear factor kappa B (NF-κB) ligand (RANKL), which in turn causes undifferentiated osteocytes to develop into osteoclasts.

## 4. Apoptosis Research Using the HS Rat Model 

HS is used to simulate microgravity and mimic its effects on the musculoskeletal [49,50,51,52,53,54], immune [55,56,57], and nervous [58] systems, as well as on gene expression [59]. Aguirre et al. [53] showed that the unloading of bones leads to temporally and spatially correlated osteocyte apoptosis, which somehow recruits osteoclasts. Later, researchers discovered that RANKL expression plays a crucial role in bone homeostasis [60]. Plotkin et al. [54] demonstrated that suppressing apoptosis in osteocytes and osteoblasts, and reduced RANKL expression, is not sufficient to completely inhibit the loss of bone in lumbar vertebrae. By contrast, there was no bone loss in femora upon inhibition of osteocyte apoptosis via caspase inhibitors compared with the control [49]. This discrepancy might be caused by the use of different inhibitors or the different bones analyzed.

The adaptation of muscle size to force demand is dependent on merging myoblasts and the resulting number of myonuclei [61]. During HS, myonuclei undergo apoptosis. This can be counteracted by dietary supplementation of the antioxidant carotenoid astaxanthin, which possibly counteracts the disuse-induced production of reactive oxygen species (ROS) and, therefore, ROS-triggered apoptosis [50]. Tetramethylpyrazine, a compound found in a traditional Chinese herbs and many other foods, shows a significant protective effect against HS-induced muscle weight and size loss [51]. It reduces the level of intracellular calcium, Bax, Bcl-2, and cytochrome *c* release, thereby suppressing the mitochondrial apoptosis. Farley et al. [52] recently published a paper on a study that combined HS and simulated cosmic irradiation (HSIR). The results were quite mixed. Only some parameters revealed a stronger inhibition with radiation (e.g., in the cortical bone). Sometimes, the difference between control and HS was not significant. It is important to note that this study deviated purposefully from the usual protocol for HS studies, where all animals (HS and control) are kept at room temperature (if any information is given at all). In this study, all animals were kept at 28 °C because the authors realized that the HS mounting keeps the animals from curling up at night (different from the control group), which they suggest is an important difference in terms of temperature homeostasis and, consequently, energy consumption.

In a study on immune effects, after 14 days of HS, there was a reduced number of medullary thymic epithelial cells and diminished expression of autoimmune regulator [55]; these findings suggest that HS impacts the thymic T-cell repertoire. However, there was no selective decrease in CD4^+^CD8^+^DP cells, which is in line with the findings of another study of short-term (three-day) HS [57]. Interestingly, after three weeks of HS, the increase in plasma corticosterone reported in the two aforementioned studies was no longer observed [56]. B-cells were drastically reduced, while T cells remained unchanged in number, although the Th/Tc ratio decreased.

Studying the influence of HS on the nervous system, Wise et al. [58] found elevated levels of ROS and a lack of reduced glutathione (GSH) in the brain stem and frontal cortex. Even though they did not explicitly focus on apoptosis, they hypothesised that this change might also trigger apoptosis.

Kulikova et al. [59] compared the gene expression of antiapoptotic genes, among others, in mouse brains during spaceflight and HS to their respective controls. They found that the expression of the *Bcl2l1* gene was reduced in the striatum and the hypothalamus in spaceflight, while it was unaltered after HS and elevated in the hippocampus in both conditions. The study findings were rather inconclusive, as only four out of 14 genes showed a different expression after HS, while all 14 showed different expression in spaceflight condition. 

## 5. Murine Experiments in the Space Environment

Space exposure impairs human health on several levels. Effects on the skeletal [62], nervous [63], and cardiovascular [64] systems have been described. To elucidate the effects of spaceflight on human health, more research using mammalian model systems is required. Similar to standard laboratory research, mice and rats are also preferred model systems in space research and play an important role in spaceflight programmes. For space experiments, researchers have developed highly sophisticated self-contained rodent habitats, such as the Mice Drawer System (MDS) [65] or the Animal Enclosure Module (AEM) [66]. 

It is estimated that about 30% of astronauts suffer from eye problems (e.g., cataracts) after space missions [67]. Studies have shown necrotic nuclei in eyes of rats exposed to outer space for 20 days [68]. Mao et al. [69] performed a profound study on the effects of the spaceflight environment on ocular tissues of mice flown for 13 days during a space shuttle mission (STS-135). They found that space exposure induced apoptosis in the inner and ganglion cell layers in mice. Apoptosis was most likely induced by oxidative stress and damage to the mitochondria. There was increased activated caspase-3, significant apoptotic events (denoted by a marked increase in terminal deoxynucleotidyl transferase-mediated nick end-labeling (TUNEL)-positive objects), lipid peroxidation, and modulation of gene expression of proteins involved in mitochondria-associated apoptotic pathways. Oxidative damage during space exposure has also been reported in the mouse brain [70] and skin [71]. Mao et al. [70] observed oxidative damage in the brain cortex and hippocampus due to the increase in oxidative stress biomarkers (e.g., 4-hydroxynonenal (4-HNE)-modified proteins and nicotinamide adenine dinucleotide phosphate oxidase 2 (NOX2)) and significant decrease in superoxide dismutase (SOD) expression in mice subjected to a ground-based model for spaceflight, which included low-dose gamma radiation and prolonged unloading (using HS). Researchers investigated spaceflight condition–induced biological damage in skin cells in mice flown in the STS-135 mission using the AEM [71]. After spaceflight, the expression of oxidative stress and extracellular matrix-associated genes and proteins were significantly altered in the flight group compared with the AEM control. Among 332 named biochemicals, 19 were found to be different between spaceflight skin and AEM ground control samples, including amino acids, carbohydrate metabolism, cell signaling, and transmethylation pathways of skin tissue [71]. Taken together, these data revealed that the spaceflight condition caused a shift in biological and metabolic homeostasis due to the increased regulation in cellular antioxidants, ROS production, and tissue remodeling, indicating that astronauts may be at increased risk for pathophysiologic damage or carcinogenesis in cutaneous tissue.

In another experiment during the STS-135 mission, researchers examined thymuses and spleens of flight mice [5]; they investigated anatomy, morphology, gene expression, and DNA fragmentation in T cells and cancer cells. The study revealed that a short-term mission in space resulted in significant impact in both organs: Altered expression of genes related to T cells (e.g., *Il10*, *Il18bp*, *Il18r1*, *Spp1*, *Ccl7*, and *IL6*) and cancer cells (e.g., *Casp8*, *Fgfr2*, *Figf*, *Hgf*, *Igf1*, *Itga4*, *Ncam1*, *Pdgfa*, *Pik3r1*, *Serpinb2*, *Sykb*, *Cdc25a*, *E2f1*, *Mmp9*, and *Myc*). During a recent spaceflight study (35-day exposure on the ISS), researchers investigated the microgravity effects on the thymus of mice [72]. There were significant changes in gene expression, DNA fragmentation, as well as in thymus size compared with control mice, which encountered artificial 1 *g* acceleration by centrifugation on the ISS [72]. The authors found a strong reduction in genes involved in the regulation of the cell cycle. After a 35-day exposure to the space environment, there were no signs of elevated apoptosis. The researchers speculated that apoptosis may play an initial role in thymus shrinkage, while later inhibition of cell proliferation is mainly responsible for the observed decrease in size. 

The TUNEL assay of lung cells of mice after spaceflight revealed apoptotic events along with significant changes in gene expression of proteins involved in extracellular matrix proteins and stem cell signaling [73]. However, there were no signs of necrosis, inflammation, or other destructive processes in the microscopic analysis of lung tissues. The internal pathway analysis showed that many of the differentially expressed genes clustered together, indicating a specific response of the lung to a stressful space environment. By means of gene arrays, Hammond et al. [74] analyzed the gene expression changes in kidney and liver cells of mice flown in space for 12 days. Gene set enrichment analysis revealed that both organs share similar pathways, which were changed during space exposure. Among others, genes involved in apoptosis and cell death were significantly upregulated. 

Due to the enormous amount of experience and scientific data on mice and rats, and the available infrastructure and hardware, rodents will very likely continue to play an important role as a mammalian model system for biological research in space. 

## 6. Impact of Microgravity on Apoptosis in Different Cell Types

### 6.1. Benign Specialized Cells

#### 6.1.1. Immune Cells

A spaceflight induces changes in the immune system [1]. Impairment of the immunity of astronauts even during short-term spaceflights is a recognized health risk. Lewis et al. [7] had already reported programmed cell death detected in cells grown under conditions of real microgravity in 1998. Human T-lymphoblastoid cells (Jurkat) have been studied on the Space Shuttle. After 4 h, 30% of flown cells compared with 17% of ground cells exhibited signs of apoptosis. In addition, there was a time-dependent elevation of Fas/APO-1 protein in static flown but not the in-flight 1*g* centrifuge or ground control samples [75]. In addition, Jurkat cells flown on STS-80 and STS-95 revealed an increase in apoptosis and release of the soluble form of the cell death factor, Fas/APO-1 (sFas) [76]. Data showing elevated Fas in cells of elderly persons compared with spaceflight-related findings suggest possible similarities between microgravity effects and ageing on lymphocytes [76].

A recent publication reported about the effects of Jurkat cells and their multidrug-resistant subline, Jurkat/A4, after RPM exposure [77]. The viability of RPM-treated Jurkat/A4 cells decreased compared with the Jurkat cells. RPM-exposed Jurkat cells appeared less susceptible to apoptosis than their multidrug-resistant clone Jurkat/A4 cells [77]. In addition, intercellular adhesion molecule 3 was a key factor involved in the induction of leukocyte apoptosis [77].

Another group studied the effect of simulated microgravity using a National Aeronautics and Space Administration (NASA)-developed rotary cell culture system (RCCS) on human peripheral blood mononuclear cells (PBMCs). The level of secreted TNF-α was significantly elevated in PBMCs [78]. Researchers also noted a similar outcome in human lymphocytes: The lymphocytes exhibited increases in apoptosis induced by simulated microgravity created by an RPM [79]. Programmed cell death in lymphocytes is caused by a mechanism based on calcium-dependent 5-LOX activation, damage of the mitochondrial membrane, the release of cytochrome *c* and caspase activation [79]. Nine years later, Maccarrone et al. [80] confirmed the results obtained with the help of the microgravity simulator in real microgravity on the ISS. The role of apoptosis in lymphocyte depression (ROALD) experiment was flown to the ISS as part of the BIO-4 mission of the ESA [80]. Real microgravity exposure of human lymphocytes for two days in space elevated apoptosis (threefold), as determined by DNA fragmentation, cleaved poly (ADP-ribose) polymerase-1 (PARP) protein content, *TP53* gene expression, calpain messenger RNA (mRNA), and an early increase in 5-LOX activity [80]. 

Furthermore, simulated microgravity using a rotating bioreactor induced apoptosis in T cells compared with the static 1 *g* control cultures [81]. The Fas/CD95 expression of T cells was enhanced by pre-exposure of the cells to simulated microgravity. It is known that the extrinsic pathway of apoptosis, in particular the CD95 pathway, is involved in cellular apoptosis in cells exposed to space conditions [76].

The effect of simulated microgravity on apoptosis in lymphocytes is controversial. There are also studies reporting about inhibition of apoptotic cell death in human lymphocytes cultured in a modelled microgravity condition [82]. The authors used the rotating wall vessel culture system [82]. Another group demonstrated a reduced expression of cell-cycle genes in lymphocytes exposed to the RWV. In addition, the authors found the downregulation of pro-apoptotic genes. The authors suggest that extended exposure to simulated microgravity may result in a reduction of the cells’ ability to undergo apoptosis [83].

The microRNA (miRNA) and mRNA expression profiles in human peripheral blood lymphocytes (PBLs) exposed to the RVW revealed 42 differentially expressed miRNAs in simulated microgravity-incubated PBLs compared with 1 *g* controls [84]. Bioinformatic evaluations showed changes in biological processes, among others, in the stress response, apoptosis, and proliferation regulation. Cell viability and apoptosis assays validated the data obtained by bioinformatic analyses: Lymphocytes cultured in simulated microgravity conditions exhibited elevated apoptosis and reduced cell proliferation [84].

Natural killer (NK) cells are large, granular, cytotoxic lymphocytes that lyse virus-infected or oncogenically transformed cells [85]. NK cells exposed to an RWV for 48 h exhibited reduced cytotoxicity, along with an elevated rate of apoptosis and necrosis [86].

By 1996, rats had been flown for 14 days in space (Spacelab Life Sciences-2 mission on STS-78) and exhibited lymphatic tissue changes such as transient apoptosis [87]. Another in vivo study investigated C57/BL6 mice flown on a 30-day space high-orbit satellite mission (BION-M1); the results confirmed the earlier findings [88]. The high-orbit spaceflight environment induced elevated levels of p53 and phosphorylated p53 proteins in thymic lymphocytes. Twelve hours after landing, the calculated phospho-p53/p53 ratio increased by more than twofold, indicating apoptosis. Seven days after landing, this ratio was further increased. In addition, caspase-3 in thymic cells indicated an increase in apoptosis. These data demonstrate that real microgravity induces an increase in apoptosis in mice that is not restored by seven days after landing [88]. 

Researchers harvested organs (thymus, spleen) from C57BL/6 flight mice (FLT) after their return from a 13-day Space Shuttle mission (STS-135) and from ground control mice. The DNA fragmentation, as measured by TUNEL staining in the thymus, was greater in the FLT group (*P* < 0.01) compared with 1 *g* controls [5]. Interestingly, pathway analyses revealed a downregulation of programmed cell death and inhibition of cell cycling. The authors concluded that because the FLT group exhibited an increase in apoptotic cells, the decreased apoptosis and cell cycling pathways in living cells indicate the efforts of the immune cells to survive. It must be considered that several factors—altered gravity, cosmic radiation, stress, and the attempt of the organism to readjust to the 1 *g* gravity after their return to Earth—may explain these findings [5].

Moreover, researchers detected apoptosis in the spleen and thymus of mice exposed to HS [89]. These results were similar to the findings detected after spaceflight. The researchers visualised DNA breaks by TUNEL staining in splenocytes and thymocytes [89]. Osteopontin (OPN) is involved in cell survival and has been implicated in the process of programmed cell death in various disorders [90]. 

Using the HS model, Wang et al. [57] compared OPN knockout (OPN^−/−^) mice with wild type (OPN^+/+^) mice subjected to HS for three days. The authors detected an increase in apoptotic splenocytes and thymocytes in HS OPN^+/+^ but not in HS OPN^−/−^ mice or control mice. Lymphocytes from both OPN^−/−^ and OPN^+/+^ mice were similar with regard to their reaction to corticosteroid stimulation [57]. Immune cells in OPN^−/−^ mice seem to be protected from stress-induced apoptosis. The authors concluded that OPN seems to be important for immune cell apoptosis in mice exposed to HS and thus to external stress produced by HS [57].

Microgravity and cosmic radiation are common in space and both influence the immune system of humans (not only) in space. Human B lymphoblast HMy2.CIR cells were exposed to an RWV bioreactor for 30 min and to heavy carbon ion irradiation [4]. Simulated microgravity decreased heavy ion radiation–induced cell survival and increased apoptosis in HMy2.CIR cells [4]. 

In summary, there is evidence that exposure of lymphocytes to spaceflight as well as to simulated altered gravity conditions created by microgravity-simulating devices results in elevated apoptosis of immune cells.

#### 6.1.2. (Cardio)Vascular System

Cardiovascular disease caused by endothelial dysfunction is known to occur in astronauts after spaceflight [91]. At the cellular level, cardiovascular disease may correlate with the microgravity-induced impact on endothelial cell (EC) proliferation, survival, and apoptosis. However, these effects are likely EC-type specific. During the exposure to microgravity, human umbilical vein endothelial cells (HUVECs) [92,93] and bovine aortic endothelial cells (BAECs) [94] have been found to proliferate faster without increased signs of apoptosis, whereas microvascular ECs (HMECs) [95,96] and other ECs [11,97] showed inhibited growth or were directly induced to undergo apoptosis. Pan et al. [98] demonstrated that a specific miRNA (miR-27b-5p) could protect HUVECs from apoptosis during simulated microgravity by inhibiting zinc fingers and homeoboxes protein 1 (ZHX1). Recent studies also suggested that autophagy and mitophagy play a protective role against ER stress-mediated apoptosis in simulated-microgravity-exposed HUVECs [99,100]. However, Xu et al. [101] reported that HUVECs showed increased apoptosis after the return to normal gravity. A possible explanation could be the expression of transcription factors, such as serum response factor (SRF) and mammalian target of rapamycin (mTOR), which are critical regulators in vascular ECs and are associated with endothelial dysfunction. Both factors were altered during simulated microgravity exposure [101].

In pulmonary HMECs, researchers explained the increased apoptosis during culture in simulated microgravity by elevated NF-κB expression, downregulation of the phosphoinositide 3-kinase (PI3K)/AKT pathway, and F-actin depolymerisation [102]. Another study showed that miR-503-5p is involved in the induction of apoptosis, at least in part, by inhibiting the expression of the anti-apoptotic protein Bcl-2 under simulated microgravity conditions [103]. On the contrary, there was no apoptosis when dermal HMECs (and HUVECs) were cultured in the RWV or on the RPM [96]. This could be explained by the rapid induction of heat shock protein 70 (HSP70), which protects ECs from apoptotic stimuli by acting downstream of cytochrome *c* release and upstream of caspase-3 [92,104,105]. 

Increased apoptosis after RPM exposure has also been observed in the porcine aortic endothelial cells (PAECs) [97] and the endothelial EA.hy926 cell line created by fusing HUVECs with carcinomic human alveolar basal epithelial cells [11]. Morbidelli et al. [97] reported that simulated microgravity modified the gene expression pattern of PAECs, a phenomenon that triggered proapoptotic signals. They found an upregulation of *TP53*, *FASLG*, and *BAX* genes together with a downregulation of *BCL2* and *PCNA* genes. In addition, mitochondrial disassembly suggested the activation and involvement of the intrinsic pathways of apoptosis [97]. In EA.hy926 cells cultured on an RPM, Infanger et al. [11] found further signs of apoptosis such as caspase-3 activation and elevated PARP cleavage. Interestingly, Vascular endothelial growth factor supplementation seemed to have a cell-protective influence on EA.hy926 cells exposed to simulated microgravity [11]. However, when EA.hy926 cells are cultured in microgravity, not all cells are induced to undergo apoptosis. Thus, researchers have investigating 3D-tube formation during long-term microgravity experiments both on an RPM [106] and in space [28,107], offering new approaches for endothelial tissue engineering. To date, it has not been clarified how EC apoptosis is triggered by microgravity and if apoptotic pathways are regulated when cells adapt to the microgravity environment.

#### 6.1.3. Brain and Eye Cells 

Disturbances of visual function are considered to be a major complication during spaceflights on the ISS and after return to Earth. Notably, up to 60% of astronauts conducting long-term endeavours (approximately six months) on the ISS and 29% of astronauts on short-duration space shuttle flights (approximately two weeks) are described to have experienced a wide spectrum of ocular responses to extended microgravity exposure, resulting in deprivation in near and distant visual acuity [67]. Physiologic and pathologic alterations associated with the microgravity environment have been studied extensively during the last two decades. However, only limited data on the response of biological systems to reduced gravity have been obtained in cellular and animal studies. To protect vision in space, NASA has declared the eye as a key focus area. Consequently, the ISS is now being used as an optimal environment to study spaceflight-induced changes, and recent findings from these efforts have shed light on the effect of this environment on the eye and brain.

Previous studies in rodents have shown that spaceflights as well as simulated microgravity induce changes in retinal structure and function. Hence, by 2006, Tombran-Tink and Barnstable [108] showed spaceflight-induced degeneration in the retina of rat neonates. Following a nine-day space mission, the researchers obtained retinal sections from at least three neonatal rats at three different stages of development and examined the histological appearance. Of note, there was loss of photoreceptor outer segments and disruption of the retina pigment epithelium (RPE) monolayer in the retinas of neonatal rodents exposed to the space environment at all three postnatal days. In addition, extensive damage to the ganglion cell layer was apparent in the spaceflight animals. Other findings suggest that microgravity increases intercranial pressure, induces vascular alterations in the eye [109], and promotes apoptosis in astrocytes [110] and photoreceptor cells in spaceflight mice compared with ground-control mice [111]. In addition, Cingolani et al. [112] showed retinal degeneration from oxidative damage, and two other studies demonstrated that simulated microgravity induces damage, including cytoskeletal alterations and changes in gene expression, in human RPE [113,114].

In a more recent report, Mao et al. [69] investigated whether spaceflight environment–associated retinal damage might be related to oxidative stress–induced mitochondrial apoptosis. Changes in the expression of genes involved in oxidative stress as well as endothelial and mitochondrial cell biology were scrutinised in female C57BL/6 mice exposed to microgravity during a 13-day mission. This study clearly showed the changes in the expression of numerous genes involved in the regulation of the mitochondria-associated apoptotic pathway in spaceflight mice retinal tissues compared with the ground control. Similarly, the expression of several genes responsible for regulating the production of ROS was significantly upregulated in the retina after spaceflight compared with ground control mice. In support of these findings, there were elevated levels of 4-HNE-modified proteins (an oxidative specific marker for lipid peroxidation) in the spaceflight samples compared with controls. The study also demonstrated that spaceflight conditions significantly increase apoptosis in the inner nuclear layer and the ganglion cell layer of the retina. Consequently, the authors indicated that astronauts may be at increased risk for late retinal degeneration in space missions [69].

Stimulated by these findings, the authors of a recent study based on biochemical and proteomic analysis set out to investigate the impact of spaceflight and artificial gravity [115]. In this study, nine-week-old male C57BL/6 mice were flown to the ISS for a 35-day mission. The flight mice were maintained either under microgravity conditions or in a so-called centrifugal habitat unit producing 1*g* artificial gravity. Microgravity significantly increased apoptosis in the mice exposed to microgravity compared with all other groups [115]. The proteomic assessment showed that several important pathways accountable for metabolic stress, apoptosis, and inflammation were significantly changed in the microgravity group compared with ground controls. These retinal cellular responses may potentially affect the blood–retina barrier integrity and visual acuity and hence underscore the impact of microgravity on the development of late retinal degeneration. Moreover, the authors noted that there were more significant alterations in regulated protein expression in the microgravity group compared to the microgravity group subjected to 1*g* artificial gravity. Thus, another big takeaway from this study is that the artificial gravity delivered some protection against the spaceflight-induced changes in the retina [115].

In another search of countermeasures to prevent retinal lesions elicited by microgravity, Lulli et al. [116] exploited the antiapoptotic activity of coenzyme Q10 (CoQ10). Previous data have shown that CoQ10, by its ability to hinder mitochondrial depolarisation, effectively inhibits apoptosis of corneal keratocytes subjected to laser-based irradiation [117,118]. Furthermore, results from ground experiments showed that CoQ10 prevents simulated microgravity–induced apoptosis and cytoskeleton alterations in human RPE (ARPE-19) cells [116]. In an ongoing study, ARPE-19 cells treated with or without CoQ10 were subjected to the space environment onboard the ISS for 72 h. The results from this study, including the assessment of the apoptosis rate, cytoskeletal morphology, telomere integrity and length, and the whole sequence analysis of RNA and DNA, are yet to be published. These findings may qualify CoQ10 as an effective countermeasure and could potentially provide a great impact on the treatment of severe eye diseases in which apoptosis is a driving force in the pathogenesis.

Neurophysiological problems have also been reported to appear in astronauts conducting spaceflights [119,120,121], suggesting that brain cells are also affected by the microgravity environment. Almost two decades ago, Uva et al. [122] conducted one of the first studies to investigate microgravity-induced alterations in cells of the nervous system. Rat C6 glioma cells were subjected to a variable duration of simulated microgravity using a clinostat. After 30 min at simulated microgravity, there were signs of programmed cell death, including chromatin condensation and nuclear fragmentation. Concomitantly, the researchers identified caspase-7 in the cytoplasm and TUNEL analysis revealed widespread DNA fragmentation. Interestingly, after 32-h incubation at simulated microgravity, the number of apoptotic cells declined significantly, suggesting that glial cells have the ability to acclimatise to reduced gravity. 

The effect of altered gravity has also been investigated in primary neural cultures obtained from the brain cortex of 17-day-old mouse fetuses [123]. To simulate microgravity, cells were incubated on an RPM for 0.5, 2, 6, or 24 h, or 5 days. Exposure to simulated microgravity resulted in a time-dependent reduction of neurite length and soma size compared with 1*g* controls. In addition, analysis of the percentage of fragmented nuclei revealed a significant increase in apoptotic nuclei 6 and 24 h after exposure to simulated microgravity. Of interest, removal of neurons from simulated microgravity for 24 h was sufficient to recover the soma size, whereas neurite length was not re-adapted to normal ground conditions; this finding underscores the possible health risk astronauts might experience during and after spaceflights [123]. 

In support of these findings, Zhao et al. [124] showed that simulated microgravity inhibited proliferation while concomitantly inducing apoptosis in malignant glioma cells (U251MG). The identified effects seem to be facilitated by the stimulation of apoptosis-associated protein (p21) expression and inhibition of insulin-like growth factor binding protein-2. 

Researchers used a 30-day flight of the Russian Bion-M1 biosatellite to investigate the impact of microgravity on gene expression of pro- and anti-apoptotic factors in male C57BL/6 mice [125]. The mice were noticeably less active compared with the ground control mice. Specifically, they presented signs of distinct inadaptation to 1 *g*. Gene expression analysis revealed reduced expression of the anti-apoptotic Bcl-xL gene in the hypothalamus and striatum. However, there were no alterations in the pro-apoptotic BAX gene in all of the investigated brain structures. Similarly, the expression levels of the trophic factor BDNF and its receptor was unaltered [125]. In a follow-up paper, the authors suggested that an imbalance between pro- and anti-apoptotic factors might explain the observed behavioural abnormalities after the 30-day spaceflight [126]. Furthermore, there was an increase in Bcl-xL expression in the hippocampus, which might indicate that this gene plays a compensatory role during long-term spaceflights.

#### 6.1.4. Chondrocytes and Bone Cells

The impact of microgravity on bone in humans, including changes in calcium, and bone metabolism, mechanisms of bone loss in space, and available countermeasures applied in space, have been studied extensively and reviewed comprehensively in a recent paper [127]. To date, research has documented that microgravity causes early-onset osteoporosis in many astronauts, resulting from massive alterations in bone tissues and a significant reduction in bone mass (reviewed in [128]). More than two decades ago, researchers hypothesised that the reduction in bone formation observed under microgravity conditions may be explained by the cephalic fluid shift, which affects bone blood flow. In 2007, authors postulated, to our knowledge for the first time, that apoptosis induced by microgravity could account for the loss of bone mass [129]. This hypothesis has been supported by several papers, all suggesting that the cellular response to microgravity alters the expression of apoptotic and anti-apoptotic genes [130,131,132,133,134,135,136,137,138,139]. As highlighted by Chatziravdeli et al. [140], several apoptotic genes, including *p53* and *BAX*, were upregulated in osteoblasts under microgravity conditions. Of note, as a response to the upregulation of these two apoptotic genes, there was a concomitant increase in anti-apoptotic genes, such as *CDKN1A*, *BCL2*, and X-linked inhibitor of apoptosis *(XIAP*) [140]. The researchers obtained these data in normal human osteoblastic (HOB) cells from trabecular bone [137]. Following incubation on a clinostat for 12–96 h, the authors investigated DNA fragmentation and mRNA levels for *BAX*, *BCL2*, *XIAP*, and *CASP3* genes in HOBs. The Bax/Bcl-2 ratio was significantly increased in samples from HOBs incubated for 24 h under simulated microgravity condition compared with 1*g* controls, suggesting an increase in apoptosis in the treated cells. Concomitantly, the level of *XIAP* mRNA was also high [137]. However, the *CASP3* level in simulated microgravity samples was similar to the 1 *g* controls. In another study by Blaber et al. [130], 16-week-old female C57BL/6J mice were exposed to microgravity conditions during a 15-day space shuttle mission. The authors reported a more than threefold upregulation in p21 expression in osteoblasts; this change suggests apoptosis resistance in these cells. At the same time, several genes related to the induction of apoptosis, including *Fbxo3*, *DapK1*, and *Cradd*, were upregulated in the flight samples [130]. However, the expression of *TP53*, which also promotes apoptosis, was slightly downregulated, suggesting that apoptosis is generally absent in short-term spaceflight mission [130].

The impact of microgravity has also been investigated intensely in chondrocytes [141,142,143,144,145]. Given that these cells are the main cellular component of articular cartilage, which has only a limited potential to heal, scaffold-free growth of chondrocytes in microgravity to form 3D aggregates for the replacement of damaged cartilage is an intriguing idea. The biology of chondrocytes, including studies involving apoptosis, has therefore been examined during short-term microgravity exposure to simulated and real microgravity conditions. Using an RPM, Ulbrich et al. [146] showed that neither caspase-3 nor Fas proteins could be detected in human tip joint chondrocytes after 24-h exposure to simulated microgravity. Similarly, only a low amount of Bcl-2 and p53 could be detected. On the contrary, Bax was expressed in significant quantities following exposure to simulated microgravity, but the level was decreased compared with the 1 *g* samples [146]. To investigate apoptosis independently of caspases and Fas proteins, researchers evaluated the level of Annexin V by flow cytometry. Using this method, they found that up to 18% of the 1*g* control chondrocytes displayed signs of apoptosis after 24 h of culturing. By contrast, about 10% of the chondrocytes exposed to simulated microgravity showed signs of apoptosis. This finding nicely demonstrates that apoptosis in chondrocytes exposed to short-term microgravity progresses via a caspase-3- and Fas-independent pathway [146]. These data support the findings of Whiteman et al. [147] showing that peroxynitrate-induced apoptosis in chondrocytes is also initiated by the alternative apoptotic pathway. The authors also noted that the alternative apoptotic pathway in chondrocytes subjected to microgravity may include Bax but not p53 and Bcl-2 [146]. These findings suggest that chondrocytes have a greater ability to resist the stress evolving during the transition from gravity to microgravity. Other researchers obtained similar results by studying chondrogenesis in rotating systems [143].

In a recent paper, the researchers investigated the gene expression of human chondrocytes cultured under real microgravity conditions during a parabolic flight campaign [145]. The viability of the cells was not affected by the parabolic flight, and in support of the studies carried out in simulated microgravity, there was a significant increase in the expression of anti-apoptotic genes. In addition, the anti-apoptotic interleukin-8 (IL-8) together with CCNA2, a cyclin exerting an important role in proliferation and cell cycling, displayed expression profiles supporting growth and cell survival following short-term exposure to microgravity [145]. Collectively, the findings obtained under short-term simulated and real microgravity showed the robustness of chondrocytes to adapt to reduced gravity. Notably, it seems that chondrocytes may actually benefit from exposure to this new environment by reducing their apoptotic rate. However, the significance of this effect needs to be investigated during long-term exposure to reduced gravity.

#### 6.1.5. Gastrointestinal, Gastric, Liver, and Pancreatic Cells

Alterations in growth patterns of benign and malignant cells are a common theme when grown under simulated and real microgravity conditions. Changes in rates of proliferation and apoptosis have been demonstrated in a number of cell types exposed to microgravity, including cancer stem cells [148] and human follicular thyroid carcinoma cells [6]. Researchers observed an inhibition of proliferation and an increase in the apoptosis index alongside morphological changes in the human carcinoma cell line SGC-7901 after 72-h incubation in a simulated microgravity condition (rotating clinostat). There was a similar trend in HFE-145 cells after 12-h incubation: Increased apoptosis that returned to baseline after 12 h; this finding indicate that the normal cell line adapted to the microgravity [149]. Chen et al. [150] identified differentially regulated metabolites related to proliferation and apoptosis in HGC-27 gastric cancer cells cultured in a rotary cell culture system. There was specific regulation of the lipid metabolism with increased levels of primary membrane phospholipids in cells exposed to weightlessness. High levels of phosphatidylethanolamine and phosphatidyl choline, indicatives of membrane proliferation, have been linked to the increased levels of apoptosis in HGC-27 and other cancer cell lines under simulated microgravity [6,124,149,151,152].

A long-term HS microgravity model caused liver damage. Increased serum levels of alanine aminotransferase and aspartate aminotransferase indicated damaged hepatocytes. Swollen mitochondria and alterations in *Bax*, *Bcl2*, and *Casp3* gene expression levels indicated increased levels of apoptosis in this model [153]. Hammond et al. [74] identified common tissue responses in kidney and liver responses to spaceflight. Transcriptome analysis revealed common overexpression of genes in pathways regulating apoptosis and cell death. There was overexpression of *CDKN1A*—a gene that encodes a protein with a regulatory role in S-phase DNA replication, DNA damage repair, and apoptosis—in both liver and kidney tissues, which is in line with the findings of Blaber et al. [154]. Microgravity appears to have beneficial effects in terms of function and growth on endocrine pancreatic beta cells. Song et al. [155] studied rat pancreatic islets cultured in a bioreactor under simulated microgravity conditions for 30 days. After seven days, the islets exhibited increased growth, higher survival rates, and decreased apoptosis compared with normal gravity control islets. Moreover, the secretory function was enhanced, as seen by the increased insulin levels from 7 to 30 days as well as morphological features, such as nutritional channels between islets and well-developed mitochondria that resembled that of islets in vivo [155]. In a later study, researchers studied pancreatic islets under simulated microgravity conditions in combination with a polyglycolic acid scaffold [156]. There was a prolonged survival time and increased insulin production and release, findings that indicate a better metabolic environment for in vitro cultured islets in simulated microgravity. Interferon (IFN)-γ, IL-1β, and TNF-α production is associated with apoptosis in beta cells in type 1 diabetes. Reduced immunogenicity and altered/reduced cytokine expression have been demonstrated under microgravity conditions and may contribute to the beneficial effects of microgravity observed in pancreatic islets [157,158].

### 6.2. Cancer Cells

Apoptotic cell death under microgravity conditions has attracted cancer scientists to look for new cancer therapy targets. First, it should be mentioned that most, but not all, tumor cell lines show increased apoptosis when exposed to microgravity (Table 1). In fact, previous results are very heterogeneous, which makes it difficult to draw a general conclusion on microgravity-induced apoptosis in cancer cells. Twenty years ago, Jessup et al. [159] reported that rotation appears to increase apoptosis in colorectal cancer cells and the reduction of rotation in microgravity decreased apoptosis with minimal changes in the growth factors regulating cell division. These findings suggest that shear stress can modulate apoptosis through pathways involving growth factor expression [159]. In this context, different levels of shear stress on microgravity simulation devices must also be considered when interpreting the apoptosis data from different cancer cell studies.

Cancer cell death has often been attributed to a dysfunctional cytoskeleton or cell cycle arrest caused by microgravity [160,161,162]. In addition, abnormal expression of apoptosis-related proteins (p53, Fas, Bax, p21, and Bcl-2) and tumor suppressor genes (*TP53*, *PTEN*, and *FOXO3*) has been found in cancer cells that showed increased apoptosis [6,124,151,152,161,163,164].

After 24-h clinorotation, 10% of ONCO-DG1 thyroid cancer cells entered into a Fas-dependent apoptotic pathway. However, the authors also detected the destruction and redistribution of mitochondria, microtubule disruption, and caspase-3 activation. This early study by Kossmehl et al. [151] demonstrated the activation of both extrinsic and intrinsic pathways of apoptosis in simulated microgravity.

**Table 1:** Studies reporting on apoptosis in human cancer cell lines cultured in microgravity.

Tumor/Cell Line	Microgravity Conditions	Apoptosis Findings	Ref.
**Thyroid cancer**			
ML-1	RPM, 24 h	p53↑, Fas↑, Bax↑, Bcl-2↓	[6]
FTC-133	RPM, 72 h	No apoptosis detected	[165]
ONCO-DG1	CN, 24 h	Bax↑, activated caspase-3, Bcl-2↓, TUNEL positive	[151]
**Breast cancer**			
MCF-7	RPM, 24 h	p53↑, Fas↑, Bax↑, caspase-8↑	[166]
	RPM, 48 h	No apoptosis detected	[163]
MDA-MB-231	RPM, 2 h	TUNEL negative	[167]
	RPM, 72 h	Bax↑, Bcl-2↓	[152]
	RCCS, 7 d	Bcl-2↓, MMP9↓, cyclin D3↑	[160]
**Colorectal cancer**			
DLD-1	RCCS, 48 h	PTEN↑, FOXO3↑, AKT↓	[161]
MIP-101	real microgravity, 10/12 d	EGFR↓, TGF-α↓, TGF-β↓	[159]
**Gastric cancer**			
SGC-7901	CN, 72 h	TUNEL positive	[149]
**Lung cancer**			
CRL-5889	RPM, 72 h	TUNEL positive	[164]
**Hepatoblastoma**			
HepG2	3D-CN, 72 h	*BAX*↓, *CDKN1A*↓, *PTEN*↑, *DRAM1*↑, *PRKAA1*↑	[168]
**Glioma**			
U251	CN, 72h	p21↑, IGFBP-2↓	[124]
	CN, 24 h	Bcl-2↓, Bnip3↓, cleaved caspases 3/9↑	[169]
C6	RPM, 1-24 h	Translocation of Bax, Bcl-2	[170]
**Melanoma**			
BL6-10	CN, 24 h	Bcl-2↓, Bnip3↓, caspases 3/7/8↑	[171]

CN, clinostat; RCCS, rotating cell culturing system; RPM, random positioning machine; TUNEL, terminal deoxynucleotidyl transferase-mediated nick end-labeling; ↑, upregulation; ↓, downregulation.

The balance between the pro-apoptotic Bax protein (increased in microgravity) and the anti-apoptotic Bcl-2 protein (decreased in microgravity) may explain the typical morphological changes related to apoptosis, including membrane blebbing, loss of the nuclear envelope, chromatin condensation, and cellular fragmentation into apoptotic bodies. Bax and Bcl-2 regulate cytochrome *c* release into the cytoplasm, a phenomenon that activates caspases. Zhao et al. [171] found an increase in apoptosis in simulated microgravity–grown BL6-10 melanoma cells probably caused by the downregulation of two anti-apoptotic proteins (Bcl-2 and Bnip3) and the upregulation of caspase-3, -7, and -8. Deng et al. [169] confirmed the downregulation of Bcl-2 and Bnip3 in glioma cells exposed to simulated microgravity. Moreover, they observed the upregulation of cleaved caspase-3 and -9 under simulated microgravity conditions leading to enhanced glioma cell death. In addition to expression changes, Bonfiglio et al. [170] recently reported about a transient cytoplasmic/nuclear translocation of Bax and Bcl-2 in C6 glioma cells triggered by simulated microgravity. This indicates further potential interactions of these proteins within the nuclear compartment, influencing the apoptotic cascade pathway.

The repeated upregulation of the tumor suppressors PTEN (Phosphatase and Tensin homolog) and p53 in microgravity can induce cell cycle arrest. PTEN is known to increase the number of G_1_-phase cells and, concomitantly, it reduces the number of S-phase cells, a key stage of DNA synthesis; these actions inhibit the uncontrolled proliferation of cancer cells. PTEN was upregulated in DLD1 colorectal cancer cells grown on an RCCS-HARV [161]. PTEN leads to AKT downregulation and further induction of apoptosis through the upregulation of the CDK inhibitors CDKN2B and CDKN2D. Thus, Arun et al. [161] suggested that cell death of colorectal cancer cells in simulated microgravity is caused mostly through the activation of cell cycle inhibition pathways.

p53, as a transcription factor, regulates the transcription of several cell cycle genes *(CDKN1A*, *SFN*, *GADD45A*, and *CCNB1)*, acting on the G_1_ and/or G_2_/M checkpoints. In addition, p53 is involved in the regulation of other processes leading to apoptosis induction: It can activate other apoptotic genes (*BAX*, *CDKN1A, MDM2, PMAIP1*, *BBC3*, and *PERP)*, upregulate Fas-mediated cell death, and induce different proteins that increase ROS production; these changes are the main causes for mitochondrial damage and apoptosis. However, Fukazawa et al. [168] demonstrated that caspase-3 activation induced by *cis*-diamminedichloroplatinum treatment under simulated microgravity was independent of the p53 status in HepG2 hepatoblastoma cells, suggesting that enhanced apoptosis signaling under simulated microgravity conditions may be regulated through signals other than those transmitted by p53.

Chen et al. [172] recently speculated that autophagy may also be an important factor in inducing cancer cell death by promoting apoptosis. The authors described that during the early stage of cancer progression, autophagy in ML-1 and ONCO-DG1 thyroid cancer cells significantly increased compared to other cell lines. This could explain the different starting points of apoptosis in microgravity cultures: In ML-1 and ONCO-DG1 thyroid cancer cells, apoptosis increased after 24 h exposure to simulated microgravity [6,140], whereas in MDA-MB-231 breast cancer and SGC-7901 gastric adenocarcinoma cells, apoptosis increased only after 72 h [149,152]. On the other hand, the number of secondary lysosomes also increased in MDA-MB-231 cells cultured in a RCCS [160]. A possible role of miRNA expression in the regulation of autophagy-related apoptosis in microgravity needs to be investigated in future studies [172].

Apoptosis can also be reduced in cancer cells under microgravity conditions. One possible explanation could be that survivin (gene: *BIRC5*), an inhibitor of apoptosis that is overexpressed in cancer but not in normal tissues [173], is also influenced by microgravity [152,174]. While survivin’s exact mechanism of action is not yet fully understood, it may inhibit apoptosis by directly interacting with effector caspases 3 and 7, thus preventing their activation [175]. This way survivin interferes with the caspase-independent AIF (apoptose inducing factor) pathway of apoptosis [176]. In addition, survivin physically associates with cyclin-dependent kinase p34^Cdc2^ and thereby leads to the inhibition of the pro-apoptotic function of p34^Cdc2^ and enhanced cell survival.

## 7. The Impact of Cosmic Radiation on Apoptosis

An important field of interest in space research is to study the effects of space radiation on organisms. Radiation is considered one important constraint for the manned exploration of the solar system [177,178]. In space, radiation may act independently or synergistically with microgravity and other factors to produce significant effects on organisms. 

The radiation environment in space is very different from that on Earth and mainly depends on the solar activity and location in space. The main components of radiation in space are galactic cosmic radiation and solar cosmic radiation [177,178,179]. Galactic cosmic radiation, which is mainly produced by the Sun and supernova remnants, consists of 87% high-energy protons (hydrogen nuclei), 12% alpha-particles (helium nuclei), and 1% heavier nuclei (high-charge and high-energy particles (HZE—high (H) atomic number (Z) and energy (E))) [178,179,180]. The solar cosmic radiation comprises low solar wind particles to highly energetic charged particles that are emitted sporadically from the magnetically disturbed regions of the Sun (solar energy particle events) [178,180,181]. The components of solar cosmic radiation are protons (major) and alpha-particles [182]. The solar particle events mainly consist of 92% protons, 6% helium ions, and 6% HZE ions [183]. The HZE particle exposures have important implications because they have high linear energy transfer (LET) values (> 10 keV/µm) that induce more complex cellular damages (chromosome aberrations, difficult-to repair DNA damage) and overall biological effects compared with low-LET radiation of the same energy [184,185]. Complete protection from HZE particles is practically impossible and is considered to be the main contributor to radiation health risks [186,187]. 

Exposure to space cosmic radiation causes significant health risks to astronauts (acute radiation sickness, skin injury, and numerous other biological effects) in manned space missions. Therefore, it is important to understand the biological effects of cosmic radiation on humans and other organisms (animals, plants, and microbes) for interplanetary missions as well as to develop bio-regenerative life-support systems needed for such missions. Numerous experiments have been conducted on animal and cell models aboard different satellites and space stations and on Earth to study the effects of cosmic radiation on organisms from the perspective of manned space missions. Several ground-based facilities producing low dose rates, high-energy particles, and microbeam irradiation for evaluating the biological effects and health risks of ionizing radiation are available [177]. HZE particles, such as carbon and iron, are mostly used for ground-based studies. In addition to animal models, results from various studies or situations in which humans had been exposed to radiation (accidently or occupationally) have been used to generate models to determine the risk of radiation in humans during spaceflights [188,189]. However, there is still a high degree of uncertainty on radiation-induced effects. Many questions on the biological effects of cosmic radiation on cells and organisms still remain unanswered. This is due to the type and quantity of radiation encountered by cells and organisms, the tissue of choice, the biological endpoint, and the characteristics and developmental stage of an organism [188,190,191].

Space radiation exposure on long-duration spaceflights can cause cellular stress, alter cell fate decisions, increase the risk of cancer and cardiovascular diseases, lead to cataracts and tissue degeneration, and affect the central nervous system (neurological disorders, premature ageing, Alzheimer’s disease or other dementia) and immune function of astronauts [91,186,192,193,194,195,196,197]. The nature and degree (complexity) of cellular, sub-cellular, and biological changes and health risks posed by the exposure to radiation depend on the dose, rate, quality, and energy and particle flux of the radiation. The major effect of space ionizing radiation in cells is complex DNA damage (single- or double-strand breaks). DNA damage stimulates cellular responses that activate appropriate DNA repair mechanisms (base-excision repair, homologous recombination, or non-homologous end joining) depending on the nature of the damage and arrest the cell cycle to provide time for repair of the injured DNA [198]. If the DNA repair mechanisms are unsuccessful, the cell undergoes apoptosis or necrosis, differentiates prematurely, becomes senescent, or transforms to become malignant and ultimately cancerous [188]. Apoptosis eliminates cells with abnormal DNA that may be harmful to the organism. DNA repair, cell cycle arrest, and apoptosis are necessary to maintain the genomic stability of cells and prevent tumor development [199,200].

Space HZE particles may impair the abilities of astronauts to perform critical tasks during long-term space missions. Radiation leads to oxidative stress and cognitive impairment (memory loss, reduced motor function, behavioral changes, and other neural effects) [126,197,201,202,203,204]. A 30-day irradiation of mice with high-LET 56Fe beams (500 MeV/nucleon, 1.5 Gy) reduced long-term memory, suggesting that cognitive impairment in radiation-exposed mice is similar to those seen in aged mice and supporting the involvement of the cerebellum in cognition [201]. Furthermore, radiation exposure increased necrosis in cerebellar Purkinje cells, while only apoptosis was observed in granule cells. In addition, apoptosis was higher in granule cells located in close vicinity to Purkinje cells [201]. Another study showed that spatial memory is damaged in mice exposed to a ground-based model of space radiation (2.0 Gy of 500 MeV/nucleon (56)Fe beams), indicating that radiation inhibits neurogenesis and causes cognitive impairments [202]. An acute dose-dependent increase in apoptosis and ROS has been observed in X-ray-irradiated (dose rate of 4.5 Gy/min) neural precursor cells of the adult rat hippocampus [204,205]. Elevated apoptosis and ROS production persisted for 1 week and 3–4 weeks after irradiation, respectively. In addition, the radiation response was characterized by the activation of cell cycle checkpoints and an increase in p53 (or Trp53), phospho-p53, and cyclin-dependent kinase inhibitor 1A (CDKN1A, also known as p21) protein levels. The authors suggested that the increase in apoptosis and ROS may be due to the p53-dependent regulation of cell cycle control and stress-activated pathways and that oxidative stress plays a role in neurogenesis inhibition and cognitive impairment development after radiation exposure [204]. Naumenko et al. [126] examined C57BL/6J mice housed on the Russian biosatellite Bion-M1 for one month to determine the effect of long-term spaceflight on the expression of genes involved in neurogenesis (brain-derived neurotrophic factor (BDNF) and BDNF receptors TrkB and p75) and apoptosis (proapoptotic factor BAX, antiapoptotic factor Bcl-xL). They noted that the spaceflight condition did not alter BAX, BDNF, and TrkB and p75 receptors, but it did change the expression level of Bcl-xL gene (reduced in the striatum and hypothalamus, but increased in the hippocampus). However, the Bcl-xL gene was found to be differentially expressed in the striatum alone in the ground control mice. This study indicated the role of antiapoptotic factor Bcl-xL for behavioral abnormalities during space missions [126]. 

Thymocytes (lymphocytes) are highly sensitive to an environment with ionizing radiation [206,207,208]. Thymocytes undergo apoptosis within a few hours after low doses of irradiation [206]. The crypt epithelia in the small and large intestines are also radio-sensitive and show apoptosis after exposure to ionizing radiation [209,210,211]. Maximum apoptosis in the small intestine transpired within 3–6 h following exposure to 1 Gy ionizing radiation [209,210]. Radiation-induced apoptosis in the small intestine occurred mostly at positions in the crypts associated with stem cells. The frequency of apoptosis in the large intestine was lower than the small intestine and the position of apoptosis-susceptible cells varied by region [209,210]. The apoptosis yield in the thymus and intestine epithelial cells varies among groups as well as strains of mice, indicating the presence of genetic regulation of radiation-induced apoptosis [207,210,211,212,213]. Genetic analysis has shown the involvement of several genes (*Rapop1*, *Rapop2*, and *Rapop3* (radiation-induced apoptosis), *p53*, and *Bcl2*, among others) in controlling the susceptibility to radiation-induced apoptosis [208,214].

Apoptosis plays an important role in proton-induced tumor cell death [215,216,217]. Ristic-Fira et al. [215] studied the late effects of single proton irradiation on HTB63 human melanoma cells and demonstrated a dose-dependent irradiation effect on melanoma cell growth, G2/M cell cycle arrest, and the appearance of apoptotic nuclei. Irradiation of different cancer cell lines (Ca301D cells of papillomatous carcinoma of thyroid, PC3 cells of prostate adenocarcinoma, and MCF7 cells of mammary adenocarcinoma) with a dose of 10 Gy of a 26.7 MeV proton beam increased the intracellular levels of hydroxyl radicals, changed cell structures, and triggered DNA double-strand breaks, resulting in the activation of the mitochondrial pathway of apoptosis and cell cycle arrest [217]. The effects of irradiation were found to be qualitatively and quantitatively similar to the controls up to 20 Gy, but exposure to 40 Gy led to a significant increase in necrosis. Furthermore, apoptosis was lower and occurred in a temporally delayed manner in cells irradiated with X-rays [217]. Proton beam irradiation increased ROS and induced the activity of caspases in tumor cells (LLC and HepG2), resulting in apoptotic cell death [218]. In a study conducted with mice irradiated with 1 GeV protons (LET of 0.24 keV/μm) at doses from 20 to 70 cGy/min at the NASA Space Radiation Laboratory (NSRL), Brookhaven National Laboratory, there was increased expression of apoptosis-related genes and proteins (BAX, caspase-3, caspase-9, caspase-8, PARP, NF-κB1 and tumor growth factor TGF-β1, Bcl-2, and Bcl-xL) [216]. Mice treated with whole-body doses of gamma (1.1 and 7.0 Gy) and proton (1.0 and 6.4 Gy) radiation showed that the apoptotic responses varied greatly between gamma and proton radiation in a tissue- and dose-dependent manner [219]. The cell-death response in some of the analyzed tissues suggested increased apoptosis following proton irradiation compared with gamma irradiation, which is in line with previous findings [217,220,221]. Peyer’s patches and the spleen showed an increased cell death response following gamma compared with proton irradiation [219]. Both types of irradiation induced nuclear accumulation of p53, while there were no significant differences in known pro-apoptotic p53-target genes in the spleens of treated mice. However, gamma irradiation caused increased expression of two p53-dependent pro-apoptotic genes, Bcl-G and granzyme B, compared with proton irradiation, suggesting that the cell fate following gamma or proton irradiation may be context-dependent and that gamma radiation triggers a more diverse non-random stress response that mediates apoptosis partially independently of the DNA damage [219].

It is important to study the effects of ionizing radiation under microgravity conditions. A 3D clinostat-synchronized heavy-ion and x-ray irradiation system simulating space-like radiation and microgravity environment has been developed to better predict the biological effects of space cosmic radiation on cells/tissue/organisms and humans [177]. Future studies with this platform will enhance our understanding of cosmic radiation effects on cells and human health and facilitate the development of strategies for mitigating risks associated with cosmic ionizing radiation during long-term space missions [177]. 

## 8. Summary and Perspectives

Gravity has been a stimulus since the formation of organic molecules of life and during the entire evolution of living organisms. Life forms have never encountered the extreme environmental conditions of space, like weightlessness (microgravity), and increased cosmic radiation, on Earth. A high degree of molecular, physiological, and morphological changes occured in cells, tissues, and whole organisms when exposed to altered gravity conditions, which are not observed under normal 1 *g* conditions. The microgravity-induced changes in signal transduction in cell death and apoptosis gives us new insights into the underlying regulatory mechanisms of apoptosis and necrosis. There are directly applicable benefits among others in the medical field, such as cancer research, where a deep understanding of the fundamental mechanisms leading to or impeding cell death will allow us to identify medical therapeutics. While initially often considered as a high-priced, low-efficient discipline at the fringes of science, microgravity research has proven its tremendous potential in accelerating and promoting life science research. Instruments or platforms providing functional weightlessness will significantly contribute to uncover the mechanisms of cell growth, development, and death; cancer development; and immune-related diseases.

Another important reason to undergo profound space research on the cellular and organismic level is to support the long-term manned missions that explore extra-terrestrial bodies and life in our solar system and beyond. During such missions, humans will be exposed to the space environment for considerably longer periods. Current studies about the negative effects of the space environment on human health, including the immune system, bone, muscle, and nervous systems, as provided in this article, are a prerequisite for sophisticated space-exploration programmes.

## Abbreviations

2D	Two-dimensional
3D	Three-dimensional
5-LOX	Arachidonate 5-lipoxygenase
AEM	Animal Enclosure Module
Apaf-1	Apoptosis protease activating factor-1
APO-1	Apoptosis antigen 1
BAECs	Bovine aortic endothelial cells
DISC	Death-inducing signalling complex
DLR	Deutsches Zentrum für Luft- und Raumfahrt
ESA	European Space Agency
FADD	Fas-associated protein with a death domain
HDBR	Head-down bed rest
HS	Hindlimb suspension
HOB	Human osteoblastic
HUVEC	human umbilical vein endothelial cells
HZE	High (H) atomic number (Z) and energy (E)
IL	Interleukin
ISS	International Space Station
LET	Linear energy transfer
MCS	Multicellular spheroid(s)
MDA-MB231	M.D. Anderson-Metastasis Breast cancer cell line
MDS	Mice Drawer System
mRNA	messenger RNA
miRNA	microRNA
mTOR	Mammalian target of rapamycin
NASA	National Aeronautics and Space Administration
NOX2	Nicotinamide adenine dinucleotide phosphate oxidase 2
NK	Natural killer
NF-κB	Nuclear factor kappa B
OPN	Osteopontin
PARP	Poly (ADP-ribose) polymerase
PFA	Paraformaldehyde
PBLs	Peripheral blood lymphocytes
qPCR	Quantitative polymerase chain reaction
RPM	Random positioning machine
RANKL	Receptor Activator of NF-κB Ligand
RCCS-HARV	Rotational Cell Culture System-High Aspect Ratio Vessel
ROS	Reactive oxygen species
RWV	Rotating-wall vessels
RIP	Receptor-interacting protein
RPE	Retinal pigment epithelial
RPM	Random positioning machine
SRF	Serum Response Factor
SOD	Superoxide dismutase
SODD	Silencer of death domain
STS	Space transportation system
TNBC	Triple-negative breast cancer
TUNEL	Terminal deoxynucleotidyl transferase-mediated nick end-labeling
TNF	Tumor necrosis factor
TRADD	TNFR-associated protein with a death domain
TS	Tubular structure
WHO	World Health Organization
XIAP	X-linked inhibitor of apoptosis
